# Physical and Bioactive Properties of Muffins Enriched with Raspberry and Cranberry Pomace Powder: A Promising Application of Fruit By-Products Rich in Biocompounds

**DOI:** 10.1007/s11130-016-0539-4

**Published:** 2016-04-01

**Authors:** Sylwia Mildner-Szkudlarz, Joanna Bajerska, Paweł Górnaś, Dalija Segliņa, Agnieszka Pilarska, Teofil Jesionowski

**Affiliations:** Institute of Food Technology of Plant Origin, Faculty of Food Science and Nutrition, Poznań University of Life Sciences, Wojska Polskiego 28, 60-637 Poznań, Poland; Department of Human Nutrition and Hygiene, Faculty of Food Science and Nutrition, Poznań University of Life Sciences, Wojska Polskiego 28, Poznań, 60-637 Poland; Institute of Horticulture, Latvia University of Agriculture, Graudu 1, Dobele, LV-3701 Latvia; Institute of Chemical Technology and Engineering, Poznan University of Technology, Berdychowo 4, Poznań, 60-695 Poland

**Keywords:** By-products, Muffins, Phenolic compounds, Tocochromanols, Stability, Microstructure

## Abstract

The objective of this study was to determine the effects on quality of incorporating raspberry and cranberry pomaces into American-style muffins prepared under various baking conditions. The different baking conditions did not affect the texture or microstructure of the control muffins. The enhanced samples baked at 140 °C for 30 min were characterized by a harder texture than the control muffins and by a distributed protein matrix and distorted starch granules, while those baked at 240 °C for 15 min had a moist texture and showed incomplete starch gelatinization. The mean percent recovery of ellagic acid, flavonols, tocopherols, tocotrienols, and anthocyanins after baking were 156, 53, 48, 43, and 22 %, respectively. Lower baking temperature was better for ellagic acid and tocotrienols, but worse for flavonols, tocopherols, and anthocyanins. It seems that, for the enhanced samples, the intermediate baking conditions (180 °C for 20 min) guarantee the best microstructure and texture and the appropriate retention of phytochemicals in muffins.

**Electronic supplementary material:**

The online version of this article (doi:10.1007/s11130-016-0539-4) contains supplementary material, which is available to authorized users.

## Introduction

Foods containing large quantities of phytochemicals are associated with a reduced risk of human diseases such as cancer, atherosclerosis, heart disease, osteoporosis, and obesity. The protective role of these foods is partly attributed to constituents such as phenolic compounds (PCs), tocochromanols and dietary fiber (DF) [1].

The food industry generates many millions of tons *per* year of processed plant by-products, particularly in the fruit and cereal processing sectors. Fruit and cereal processing by-products are only partially valorized at different value-added levels, and are extensively treated as a waste of environmental concern, with the relevant negative effects on the overall sustainability of the food-processing industry. The treatment and disposal of processing by-products is a serious environmental problem, and new technologically viable strategies for converting them to high-value food would prevent their disposal as waste, and would remarkably increase both the sustainability and competitiveness of the worldwide food industry [2].

The results of many authors indicate that the by-products of the fruit industry are rich source of PCs and DF [2–5]. Berry pomaces, including those of raspberries and cranberries, are rich sources of antioxidants [6]. Thus, it is advisable that other potential uses of those by-products are sought, not only those that might be economically sensible, but also those that might improve the wholesomeness and variety of food products.

American-style muffins are the most popular bakery food products consumed worldwide at all economic levels. This is mainly due to their ready-to-eat nature, their availability in different varieties, and affordable cost. Of baked products generally, muffins rank third among breakfast products and attract a broad range of consumers [7]. Thus, most bakery products, especially biscuits are used as sources for incorporating various nutritionally rich ingredients for their enrichment [5, 8–10]. However, bioactive ingredients that are added to biscuits to enhance their nutritional value may adversely affect the viscoelastic properties of dough and the quality of the goods, giving smaller volume, lower sensory panel scores, and a harder texture [11]. Thus, it is necessary to consider the chemical composition of the bioactive ingredients used in biscuit, with a specific focus on the impact of added DF and PCs on dough rheology and product quality. The stability of bioactive components during the baking process should also be taken into consideration, in order to ensure their optimum retention in new products.

The objective of this research was to evaluate the effects of incorporating two amounts (10 and 20 %) of raspberry (RP) and cranberry (CP) pomace on the quality of muffins prepared at three different oven temperatures and baking times (140 °C for 30 min, 180 °C for 20 min, and 240 °C for 15 min). Muffin phenolic compounds and tocochromanol stability, as well as textural and microstructural parameters, were used as indices for comparison.

## Materials and Methods

### RP and CP Preparation

Fully mature European raspberries (*Rubus idaeus* L.) and American cranberries (*Vaccinium macrocarpon* Aiton) were pressed using a 60K basket press (Voran Maschinen, Pichl bei Wels, Austria). The pomace thus obtained was oven-dried using an Orakas 5600 (Marlemi, Lemi, Finland) with forced hot-air circulation at 55 ± 1 °C for 16 h. The dried pomace was milled to obtain a particle size of 0.5 mm, and stored at -18 °C until use. The chemical characteristics of obtained RP and CP powders are shown in Table A (ESM).

### Preparation of Muffins

The muffin formulation contained the ingredients typically used for muffin preparation: 34.05 % wheat flour; 32.13 % water; 15.42 % sugar; 13.88 % fat; 2.57 % non-fat dry milk powder; 1.29 % baking powder; 0.53 % dry egg white; and 0.13 % salt (weight basis) [7]. RP and CP pomaces were incorporated into the muffins at 0, 10, and 20 % (*w*/*w*) by replacing an equivalent amount of wheat flour in the muffin mixture. Before the experiment, a pilot baking trial of control samples without the addition of pomaces was conducted to determine the optimal temperature and baking time for well-baked muffins. Based on this, the dough (70 g) was placed into paper muffin cups and baked in a pre-heated oven at 140, 180, and 240 °C for a total baking time of 30, 20, and 15 min, respectively. After baking, the muffins were cooled to room temperature and packed in polypropylene pouches. They were then sealed until sensory and textural analysis. Other muffins intended for chemical analysis were frozen, freeze-dried, ground into a fine powder, and stored at -18 °C in airtight vials.

### Physical Characteristics of Muffins

The textural characteristics of the muffins were determined using a texture profile analysis with a TAXT2 texture analyzer (Stable Micro Systems, Surrey, UK). Square-shaped pieces of cake measuring 5 cm on all sides and with a sample thickness of 2.5 cm were evaluated by compressing them twice to 50 % of their original height. Hardness, gumminess, chewiness, and cohesiveness of the muffin crumb were measured based on the force–time curve.

The surface morphology and microstructure of the muffin samples were examined on the basis of the scanning electron microscope (SEM) images recorded using a Zeiss scanning EVO40 electron microscope. Before examination, the muffins (size 20 × 20 mm) were defatted with hexane and freeze-dried. The muffin samples were placed separately on the sample holder using double-sided adhesive tape and exposed to gold sputtering (2 min, 2 mbar). Finally, each sample was transferred to the microscope, where it was observed at 15 kV and under a 9.75 × 10^−5^ Torr vacuum.

### Tocochromanols Analysis

The tocochromanols were extracted from the pomaces and muffins according to previously developed method [12]. The chromatographic separation was carried out on a Shimadzu HPLC system (Shimadzu Corporation, Kyoto, Japan) consisting of a fluorescence detector (RF-10AXL) and a Luna PFP column (3 μm, 150 × 4.6 mm) with a guard column (4 × 3 mm) (Phenomenex, Torrance, USA). Identification and quantification were performed using a fluorescence detector at an excitation wavelength of 295 nm and an emission wavelength of 330 nm.

### Phenolic Compounds Analysis

The phenolic compounds were extracted from the pomace and muffins using a method described by Makarova et al. [13]. The polyphenol composition was determined using a Shimadzu HPLC system (Kyoto, Japan) consisting of a photodiode array detector (SPD-M20A) and an Inertsil ODS-3 column (150 mm × 4.6 mm, 5 μm) (GL Scientific, Tokyo, Japan). The mobile phase consisted of 5 % formic acid in water (A) and methanol (B), and was programmed as follows: 0 min, 10 % B; 5 min, 20 % B; 8 min, 10 % B; 15 min, 30 % B; 17 min, 40 % B; 25 min, 55 % B; 40 min, 70 % B; 45 min, 100 % B; 50 min, 10 % B. The compounds were monitored at 280 nm (flavan-3-ols, dihydrochalcones), 320 nm (hydroxycinnamic acids), 370 nm (flavone glycosides and aglycones, and ellagic acid), and 530 nm (anthocyanins). The unidentified polyphenols were collected in the end of the capillary and identified using mass spectrometry. They were quantified as follows: flavone glycosides as quercetin-3-*O*-rutinoside, and anthocyanins as cyanidin-3-*O*-rutinoside.

### Recovery Rate

The percentage recovery of polyphenolics and tocochromanols from RP and CP incorporating muffins was calculated according to Rupasinghe et al. [14].

### Statistical Analysis

Statistica software version 10.0 (StatSoft, Kraków, Poland) was used to determine whether the variables differed among treatment groups. Two-way ANOVA was applied to assess the effects of the level of RP and CP (*L*) and baking conditions (*Bc*), and the interaction between these factors (*L* × *Bc*). When the ANOVA indicated significant treatment effects, the means were evaluated using Tukey’s honestly significant difference test. The results are presented as the mean values of three analyses.

## Results and Discussion

### Physical Characteristics of Muffins

Table 1 presents the texture of the muffins prepared by replacing wheat flour with RP and CP at 10 and 20 % and baked under different conditions. Generally, the texture profile of the muffins seems to be dependent on the pomace levels (*L*, *P* < 0.001), the baking process (*Bc*, *P* < 0.001), and on their interaction. A significantly increase (*P* < 0.001) in the hardness, gumminess, and chewiness of the muffins was observed relative to the control for CP-formulated muffins baked at 140 °C for 30 min. Moreover, the samples with the addition of RP baked under the same conditions showed higher values of these indices than the control. The cohesiveness value of RP and CP muffins, regardless of baking condition, decreased compared to control. Grigelmo-Miguel et al. [15] also reported an increase in the hardness of muffins upon the addition of peach dietary fiber. The harder texture might be attributed to the dilution of gluten upon addition of the fiber fraction. The varying hardness of the enhanced samples might also be explained by the higher water absorption of fiber-rich incorporated doughs. This observation is explained by an interaction between water and the hydroxyl groups of polysaccharides through hydrogen bonding. The significantly harder texture of CP-formulated muffins, compared to the RP-formulated muffins, might be associated with a higher water holding capacity (WHC) of CP fiber (15.7 *vs*. 7.4 g H_2_O/g solid). Although the baking conditions did not affect the texture of the control muffins, they had significant effects on the enhanced samples. Interestingly, the samples with the addition of CP baked at 240 °C for 15 min had lower hardness, gumminess and chewiness values than the control, indicating a tender texture with a more compact, less aerated crumb (Table 1). The control muffins, regardless of baking condition, had the typical structure of muffins, with bubbles of different sizes and a crumb with a dry appearance, while the muffin formulations with the pomaces baked at higher temperatures showed a moist crumb structure with no appreciable large bubbles. This might be due to the water retention during baking and to fiber addition. The increase in WHC with the added fibers involves a higher amount of water loss during baking, enhancing weight loss and increasing hardness. However, at the higher temperature, the baking time was too short to evaporate all the water absorbed by the fiber, giving a moist crumb.

**Table 1:** Texture profile of muffins with the addition of raspberry (RP) and cranberry (CP) pomaces baked under different conditions

		Hardness [N]	Gumminess [N]	Chewiness [N mm]	Cohesiveness
140 °C/30min	M0	37.06 ± 6.57^c,d,e,f^	21.70 ± 3.09^d^	18.56 ± 2.55^e^	0.59 ± 0.02 ^a,b,c,d^
	RP10	44.10 ± 2.62^b,c^	24.47 ± 2.31^c,d^	21.28 ± 2.12^c,d,e^	0.55 ± 0.03 ^b,c,d^
	RP20	42.81 ± 5.10^b,c,d^	24.36 ± 2.35^c,d^	20.75 ± 2.25^c,d,e^	0.57 ± 0.03 ^a,b,c,d^
	CP10	61.54 ± 2.44^a^	32.84 ± 2.86^a,b^	28.63 ± 3.71^a,b^	0.53 ± 0.04 ^c,d^
	CP20	66.00 ± 7.50^a^	34.10 ± 4.48^a^	29.34 ± 4.90^a^	0.52 ± 0.04^d^
180 °C/20min	M0	37.15 ± 1.80^c,d,e,f^	23.10 ± 1.72^c,d^	19.60 ± 1.64^d,e^	0.62 ± 0.03^a,b^
	RP10	39.29 ± 1.55^c,d,e^	22.86 ± 1.02^c,d^	19.39 ± 0.93^d,e^	0.58 ± 0.03^a,b,c,d^
	RP20	41.37 ± 6.26^b,c,d,e^	25.50 ± 4.87^c,d^	21.27 ± 3.98^c,d,e^	0.61 ± 0.04^a,b,c^
	CP10	47.42 ± 3.88^b^	28.10 ± 2.19^b,c^	23.88 ± 2.10^b,c,d^	0.59 ± 0.04^a,b,c,d^
	CP20	60.58 ± 3.63^a^	31.58 ± 2.63^a,b^	25.70 ± 2.57^a,b,c^	0.52 ± 0.04^d^
240 °C/15min	M0	38.94 ± 1.20^c,d,e^	24.84 ± 1.34^c,d^	20.41 ± 1.18^d,e^	0.64 ± 0.03^a^
	RP10	34.49 ± 1.73^e,f,g^	20.97 ± 1.64^d,e^	18.20 ± 1.42^e,f^	0.61 ± 0.03^a,b,c^
	RP20	35.71 ± 2.93^d,e,f^	22.28 ± 2.72^d^	19.25 ± 2.53^d,e^	0.62 ± 0.03^a,b^
	CP10	27.33 ± 1.37^g^	15.66 ± 1.32^e^	12.75 ± 1.00^g^	0.57 ± 0.06^a,b,c,d^
	CP20	29.62 ± 1.94^f,g^	16.21 ± 2.59^e^	13.23 ± 2.54^f,g^	0.55 ± 0.07^b,c,d^
*L*, *P* value		<0.001	<0.001	<0.001	<0.001
*Bc*, *P* value		<0.001	<0.001	<0.001	<0.001
*P* interaction (*L* × *Bc*)		<0.001	<0.001	<0.001	NS*

Values are means ± SDs of three determinations. Within each group, in case of significant interaction, columns lacking common letter superscripts differ, *P* ≤ 0.05; *Not significant, *P* > 0.05

Sample codes: M0: control muffins; RP10 and RP20: muffins with 10 and 20 % raspberry pomace addition; CP10 and CP20: muffins with 10 and 20 % cranberry pomace addition

**Fig. 1:**
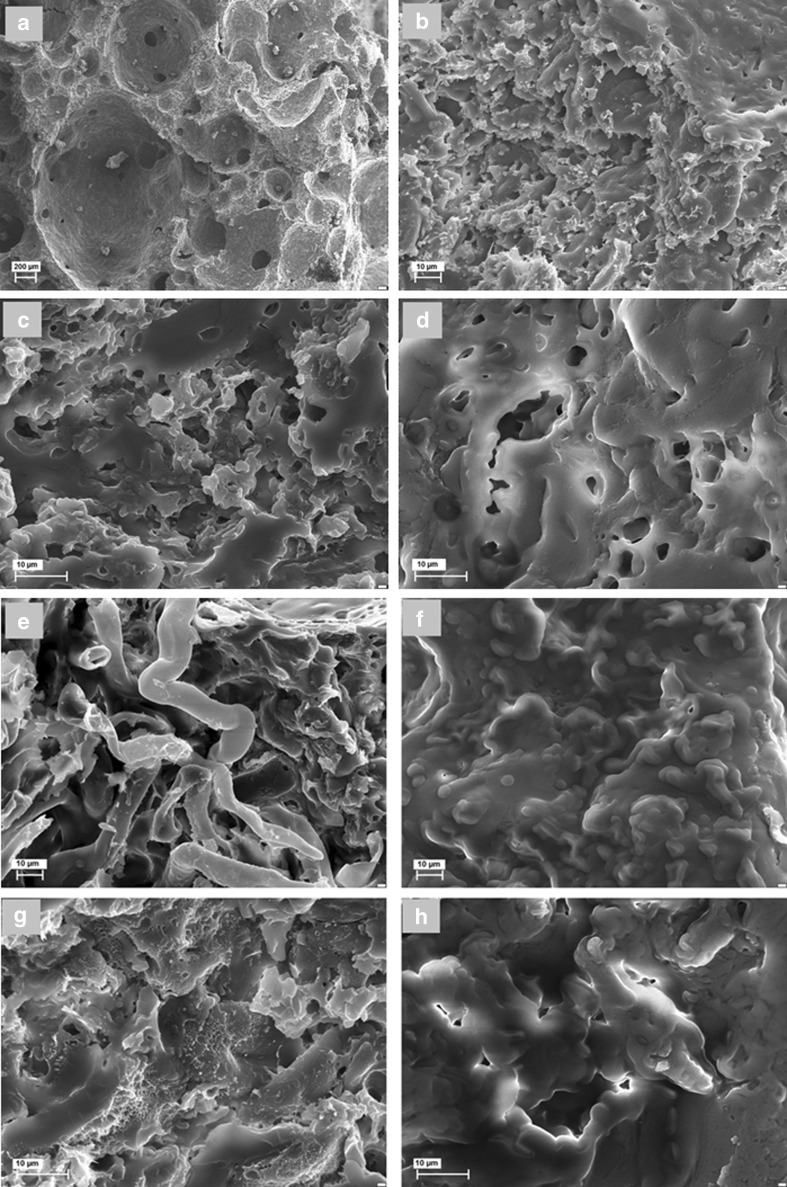
Scanning electron micrographs of muffin crumb with addition of raspberry and cranberry pomaces baked under different conditions (**a**, **b**) control muffins; (**c**) muffins with raspberry pomace baked at 140 °C for 30 min; (d) muffins with cranberry pomace baked at 140 °C for 30 min; (**e**) muffins with raspberry pomace baked at 180 °C for 20 min; (**f**) muffins with raspberry pomace baked at 240 °C for 15 min; (**g**) muffins with cranberry pomace baked at 180 °C for 20 min; (**h**) muffins with cranberry pomace baked at 240 °C for 15 min

Figure 1 presents the SEM images of the muffin microstructure prepared under different conditions. Figure 1a shows the crumb of a control muffin (baked at 140 °C for 30 min) with its characteristic gas cells, also referred to as lamellas. The magnified picture of the control muffin (Fig. 1b) shows a continuous gluten matrix with starch granules embedded in it. The gluten matrix form a firm, continuous, flexible network, which constitutes a frame for the dough. The protein component of the dough was described as a network covering the starch. The starch has also an important role in the formation of the dough structure, as a filler of the gluten matrix. Figure 1b shows the starch granules in their two forms, unaffected and gelatinized that seem to be incorporated in the protein network. The micrographs shown in Fig. 1c,d illustrate the microstructure of muffins baked with the addition of RP and CP under the same conditions as the control. The gluten matrix in these images clearly shows the presence of voids, indicating a distributed protein matrix and confirming the observation that the texture of the muffins baked with the addition of fruit pomaces (and especially of CP) changed undesirably. A similar disrupted protein matrix in the SEM images of muffins was also observed by Filipčev et al. [16] upon the addition of buckwheat and rye. However, as shown in Fig. 1c, corresponding to the RP muffins, cracks in the gluten network are much less visible than in Fig. 1d, which shows the crumb of the CP muffin. Moreover, these two SEM images (Fig. 1c,d), particularly in the case of the CP-formulated muffins, showed distorted, flat, barely visible starch granules, probably due to their restricted access to water. This somewhat new form of starch granule was also observed by Rajiv et al. [17], who aptly named it a “thin sheet.” The pictures corresponding to muffins baked at 240 °C (Fig. 1f,h) show a different microstructure from those baked at 140 °C. The muffin crumb shown in the pictures has smooth, clear-cut, locally spherical starch granules in a variety of sizes. They seem to be coated, most probably by amylopectin escaping from starch granules, that burst under the effect of high temperature. The picture clearly indicates the incomplete gelatinization of starch. In the baking process, under the high temperature conditions, water is absorbed into the starch granules. The starch granules become swollen and then burst, allowing polysaccharides to escape. It is believed that excessively short baking times prevent the starch components from immobilizing the water. The process of dough structure solidification and water evaporation did not complete, as indicated by the muffin crumb and the texture profile. In contrast, Fig. 1e and g—from the muffins baked at 180 °C—show a protein network that is typical of well-baked dough. The SEM picture of the RP muffin sample (Fig. 1e) shows perfectly shaped gluten matrix with starch granules embedded in them. On the other hand, the CP muffin crumb (Fig. 1g) is composed of a strong, continuous protein matrix with visible, natural-shaped starch granules.

### Effect of Baking on Tocochromanols

**Fig. 2:**
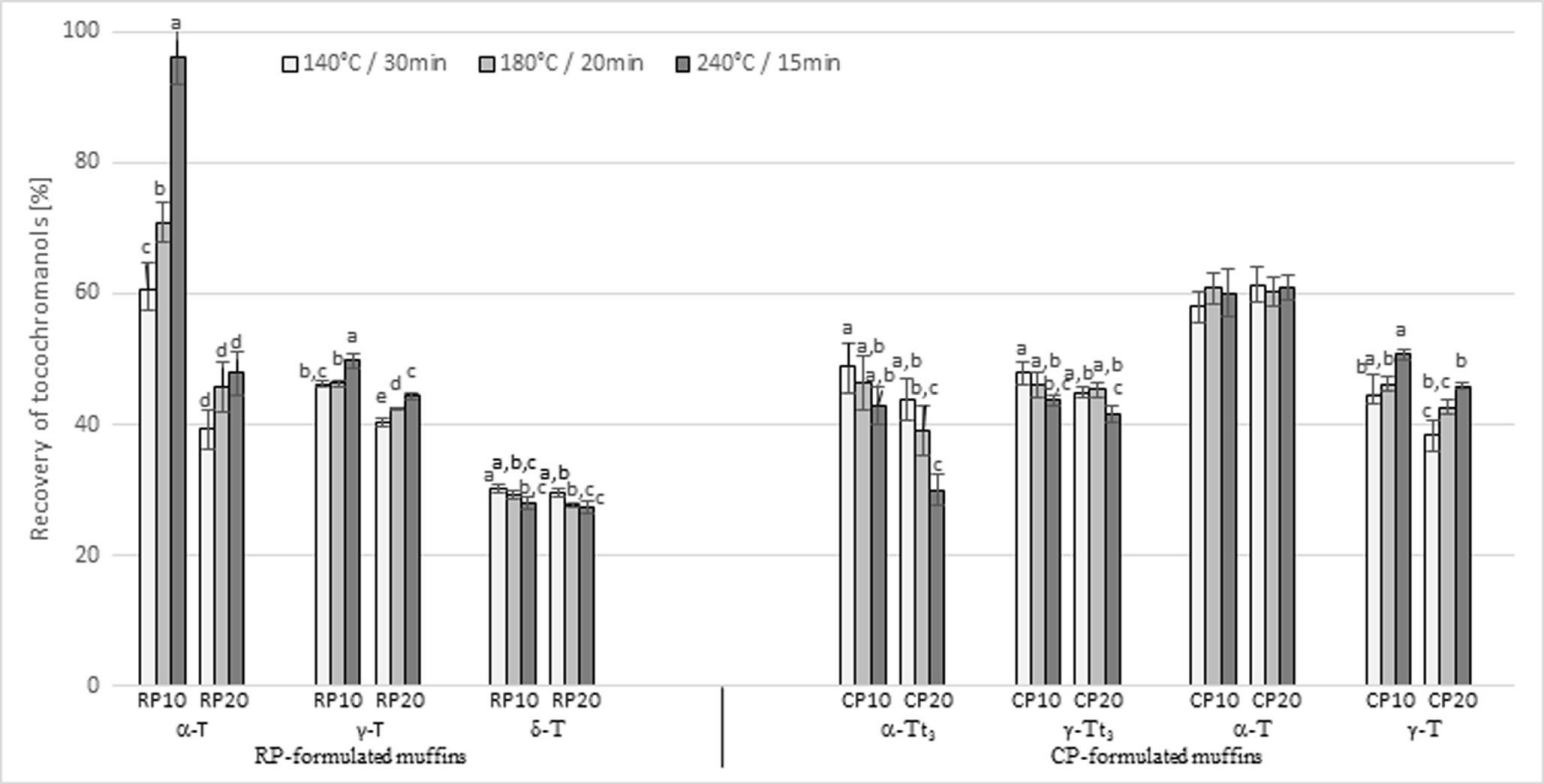
Mean percent recovery of main tocopherols (T) and tocotrienols (Tt_3_) of raspberry (RP) and cranberry (CP) pomaces after baking in a model muffin system. Values are means ± SDs of three determinations. Within each group, in case of significant interaction, columns lacking common letter superscripts differ, *P* ≤ 0.05

The RP and CP contained 66.4 and 33.5 mg/100 g dry matter (DM) of total tocochromanols, respectively (Table 2). The γ-T and δ-T were the most abundant forms in RP, while the α-T and γ-T_3_ were the most abundant forms in CP. The relatively high tocochromanol content of RP and CP pomaces could be attributed to oil from the seeds and the wax present on the skins [18]. The mean percentage of tocochromanols lost was 56 % for RP-formulated cakes and 52 % for CP-formulated cakes (Fig. 2). This is in agreement with a previous study reporting between 20 and 60 % tocopherol losses during bread and cake baking [19, 20]. The tocochromanol losses could be attributed to direct oxygenation due to the incorporation of air during mixing and to heat destruction during baking [20]. According to Hidalgo and Brandolini [19], the kneading step led to degradation of the tocopherol content, while baking had almost negligible effects. However, these authors achieved significantly lower tocopherol degradation (25.4 %) than our results, which might be due to shorter mixing times (2–3 min *vs*. 10 min in the present study). Moreover, our results suggest that both the baking conditions and the pomace levels strongly influence the tocochromanol contents of the muffins. Generally, the loss of tocopherols decreased as the temperature increased, while the loss of tocotrienols increased with the increase in baking temperature. For example, muffins made with RP baked at 240 °C were characterized by about a 27 % higher α-T recovery rate than the same samples baked at 140 °C. Conversely, α-Tt_3_ from CP had about 22 % higher stability at 140 °C than at 240 °C. However, the two-way ANOVA revealed an interaction between *Bc* and *L* only for the α-T and γ-T of the RP-formulated muffins, while the α-T of the CP-formulated muffins was not affected by all parameters (Table B, ESM).

**Table 2:** Proximate composition as tocopherol (T), tocotrienol (Tt_3_), and polyphenolic content of raspberry and cranberry pomaces

Tocopherol (T) and tocotrienol (Tt_3_) content (mg/100 g DM)			
	Raspberry pomace	Cranberry pomace	
α-T	6.37 ± 0.09	17.21 ± 0.19	
β-T	0.35 ± 0.01	0.25 ± 0.00	
γ-T	42.61 ± 1.13	1.35 ± 0.01	
δ-T	17.06 ± 0.14	0.09 ± 0.01	
α-Tt_3_	Nd	1.33 ± 0.02	
γ-Tt_3_	Nd	13.09 ± 0.12	
δ-Tt_3_	Nd	0.15 ± 0.01	
Polyphenolic content (mg/100 g DM)			
Raspberry pomace		Cranberry pomace	
Cyanidin-3-*O*-sophoroside	100.11 ± 1.81	Cyanidin 3-*O*-galactoside	263.13 ± 1.30
Cyanidin-3-*O*-glucosyl-rutinoside	38.56 ± 1.15	Cyanidin 3-*O*-arabinoside	194.14 ± 3.37
Cyanidin-3-*O*-glucoside	41.52 ± 0.42	Peonidin 3-*O*-galactoside	313.23 ± 5.06
Cyanidin-3-*O*-rutinoside	17.46 ± 0.29	Peonidin 3-*O*-arabinoside	126.95 ± 6.32
Quercetin-3-*O*-arabinoside	4.39 ± 0.31	Myricetin 3-*O*-galactoside	112.85 ± 7.86
Quercetin-3-*O*-glucuronide	6.29 ± 0.28	Myricetin 3-*O*-arabinoside	33.45 ± 2.97
Ellagic acid	30.03 ± 1.53	Quercetin 3-*O*-galactoside	124.09 ± 5.00
		Quercetin 3-*O*-glucoside	20.49 ± 0.50
		Quercetin 3-*O*-xyloside	20.19 ± 1.63
		Quercetin 3-*O*-arabinoside	21.00 ± 2.40
		Quercetin 3-*O*-rhamnoside	63.04 ± 3.29
		Syringetin 3-*O*-galactoside	25.79 ± 1.46

Values are mean ± SDs of three determinations, *Nd* not detected

### Effect of Baking on Phenolic Compounds

**Fig. 3:**
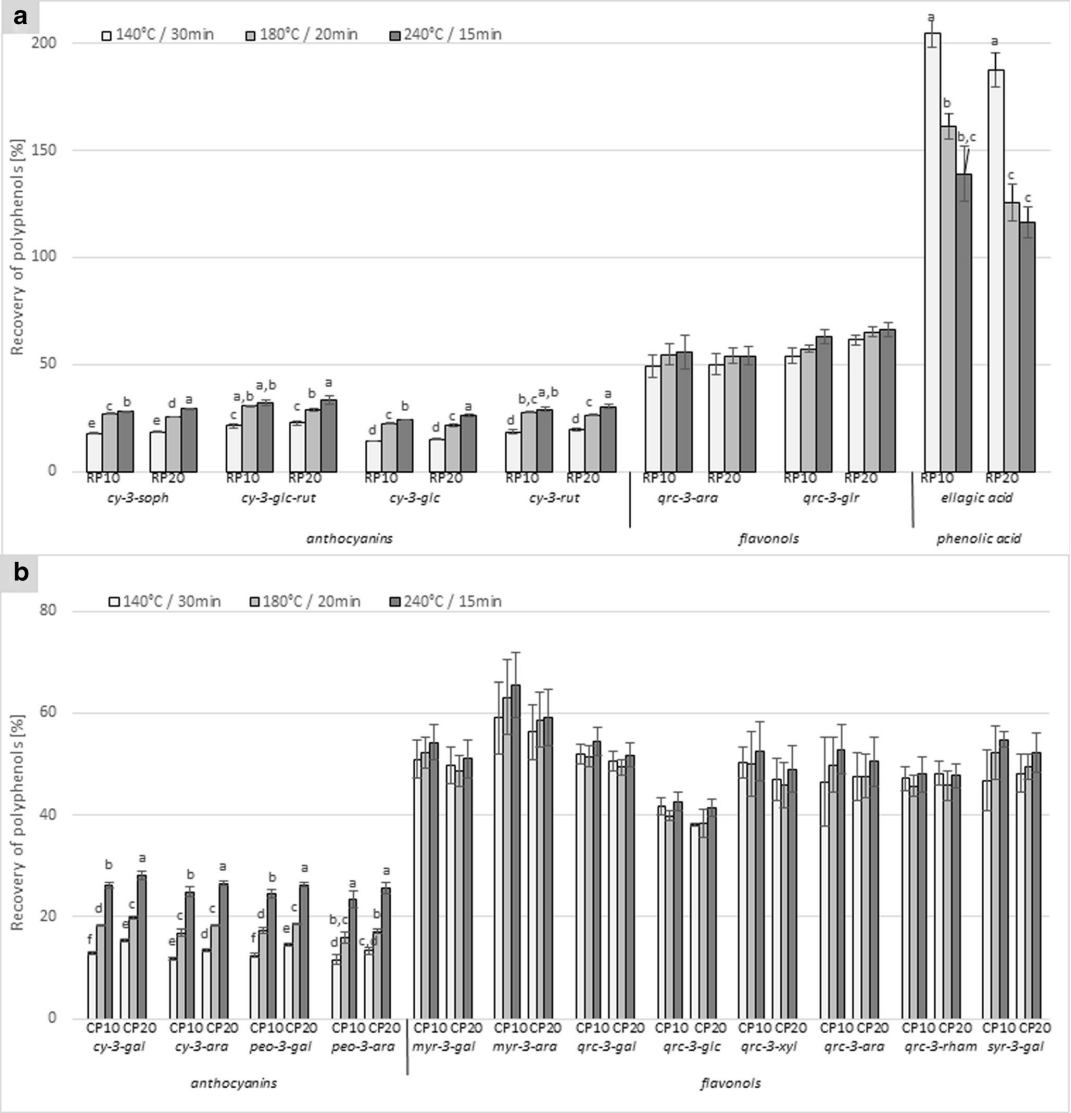
Mean percent recovery of polyphenolic compounds of raspberry (**a**) and cranberry (**b**) pomaces after baking in a model muffin system. Values are means ± SDs of three determinations. Within each group, in case of significant interaction, columns lacking common letter superscripts differ, *P* ≤ 0.05. Sample codes: M0 – control muffins, RP10 and RP20 – muffins with 10 and 20 % raspberry pomace addition, CP10 and CP20 muffins with 10 and 20 % cranberry pomace addition. Phenolic compounds: cy-3-soph – cyanidin-3-*O*-sophoroside, cy-3-glc-rut – cyanidin-3-*O*-glucosyl-rutinoside, cy-3-glc – cyanidin-3-*O*-glucoside, cy-3-rut –cyanidin-3-*O*-rutinoside, qrc-3-ara – quercetin-3-*O*-arabinoside, qrc-3-glr –quercetin-3-*O*-glucuronide, cy-3-gal – cyanidin 3-*O*-galactoside, cy-3-ara – cyanidin 3-*O*-arabinoside, peo-3-gal – peonidin 3-*O*-galactoside, peo-3-ara – peonidin 3-*O*-arabinoside, myr-3-gal – myricetin 3-*O*-galactoside, myr-3-ara – myricetin 3-*O*-arabinoside, qrc-3-gal – quercetin 3-*O*-galactoside, qrc-3-glc – quercetin 3-*O*-glucoside, qrc-3-xyl – quercetin 3-*O*-xyloside, qrc-3-rham – quercetin 3-*O*-rhamnoside, syr-3-gal – syringetin 3-*O*-galactoside

The profiles of the phenolic compounds in RP and CP pomaces were dominated by anthocyanins, which contributed to nearly 83 and 68 % of the total phenols, respectively (Table 2). Among the anthocyanins, cyanidin-3-*O*-sophoroside predominated in the RP samples, contributing to nearly 42 % of the total amount of phenolic compounds. In CP, the most abundant anthocyanin was peonidin 3-*O*-galactoside, followed by cyanidin 3-*O*-galactoside; these contributed to nearly 24 and 20 % of the total amount of polyphenols, respectively. In addition, considerable levels of flavonols were recorded in CP samples (Table 2). The CP contained a total of 420.9 mg/100 g DM flavonols. Among these, the most characteristic was quercetin 3-*O*-galactoside, followed by myricetin 3-*O*-galactoside and quercetin 3-*O*-rhamnoside. Compared to CP, the RP samples were characterized by about a 39-fold smaller amount of flavonols; however, significant amounts of ellagic acid were identified (30.0 mg/100 g DM). Based on the recovery of the original polyphenols of RP and CP from the baked muffins, the baking process affected most of them (Fig. 3). Generally, the recovery rate of polyphenols after baking under different conditions seems to be dependent on the individual compounds and their structure, which affected their thermal stability. The anthocyanins were the most affected of all the flavonols. The mean percentage losses of anthocyanins ranged from 70 to 86 % for the RP-formulated muffins and from 72 to 88 % for the CP-formulated muffins, and were about 1.7 times higher than for flavonols. Anthocyanins are not stable polyphenolics and tend to be degraded during processing [14]. However, the loss of anthocyanins significantly (*P* < 0.001) decreased in the muffins as the temperature was increased from 140 to 240 °C and baking times decreased (Table C, ESM). Anthocyanins from RP and CP were characterized by about 1.6- and 2.0 times higher recovery rates at 240 °C than at 140 °C. Despite the temperature, the stability of anthocyanins during baking also depended on the type of anthocyanin. Cyanidin-3-*O*-arabinoside and peonidin-3-*O*-arabinoside were slightly less stable in the CP-formulated muffins compared to the galactoside moieties of cyanidin and peonidins which agrees with the study of White et al. [21]. The relative stability of anthocyanins in muffins made using RP could be ranked as cyanidin-3-*O*-glucosyl-rutinoside > cyanidin-3-*O*-rutinoside > cyanidin-3-*O*-sophoroside > cyanidin-3-*O*-glucoside. This is confirmed by previous reports that rutinosides are more thermally stable than glucosides [22]. The mean percentage of loss of flavonols was 43 % for the RP-formulated muffins and 50 % for the CP-formulated muffins. Rupasinghe et al. [14] reported about 38.6 % loss of the quercetin glycosides with the incorporation of apple pomace in muffins. According to Rohn at al. [23] the main product of thermal degradation of onion quercetin glycosides is their aglycone quercetin, which remained stable during roasting at 180 °C. A slight increase in flavonol stability was observed with the increase in baking temperature but, except for quercetin-3-*O*-glucoside, there were no significant differences. According to Rohn et al. [23] the stability of the quercetin glycosides against thermal treatment ranked: 3-*O*-galactoside > 3-*O*-rutinoside > 3-*O*-glucoside > 3-*O*-rhamnoside. Our results also demonstrate that galactoside moieties have the highest thermal stability. The quercetin-3-*O*-galactoside identified in the muffins made with CP was characterized by approximately 22 % higher recovery rate than in the case of quercetin-3-*O*-glucoside. Furthermore, the present results for the two flavonols identified in RP-contained muffins indicate that quercetin-3-*O*-glucuronide had about 13 % higher stability than quercetin-3-*O*-arabinoside.

One of the most interesting finding of this study was the significant increase in ellagic acids after the baking of the samples to which RP had been added. The concentration of ellagic acid increased from the RP level of 30.0 to 298.7 mg/100 g DM (in the samples baked at 140 °C), 223.8 mg/100 g DM (in the samples baked at 180 °C) and 197.5 mg/100 g DM (in the samples baked at 240 °C) —an approximately 8-times greater content than in the case of the RP pomace. It is important to take into consideration the fact that ellagic acid is a phenolic compound strongly linked to cell walls [24]. Thus, this significant increase in ellagic acid could be related to an effect of the baking process that allowed a better release of ellagic acid from the cell wall. This observation could also be explained by the release of hexahydroxydiphenic acid from ellagitannins, which is transformed to ellagic acid [25]. Interestingly, the ellagic acid level significantly decreased (*P* < 0.001) as the temperature rose. Therefore, it is apparent that lower temperatures and longer baking times seem to enhance ellagic acid, due to the greater degradation of cell structure and its higher bioavailability; however, such processing conditions reduce the recovery rates of the other analyzed polyphenolic compounds.

## Conclusion

The chemical composition of RP and CP shows that they are good sources of phytochemicals, and could be considered as being bakery products ingredients with healthy nutritional profiles. However, it was found that RP and CP were significantly involved with the viscoelastic properties, microscopic structure, and texture profile of the muffin crumb. Moreover, baking conditions significantly influence the level of tocochromanols, leading to greater loss of tocotrienols and less loss of tocopherols with increases in baking temperature. The recovery of individual polyphenols is dependent on the individual compounds and their structures, as well as on the baking conditions. The mean percent recovery of ellagic acid, flavonols, tocopherols, tocotrienols and anthocyanins after baking were 156, 53, 48, 43, and 22 %, respectively. Lower temperature and longer baking time favored ellagic acid, due to the greater degradation of the cell structure and its higher bioavailability, but disfavored anthocyanins and flavonols. Based on these results, it seems that intermediate baking conditions (180 °C for 20 min) produce the best microstructure and texture and the optimum retention of phytochemicals in the new product.

## Electronic supplementary material

Below is the link to the electronic supplementary material.

ESM 1 (DOCX 23 kb)
