# Supplementary material for: Physical and Bioactive Properties of Muffins Enriched with Raspberry and Cranberry Pomace Powder: A Promising Application of Fruit By-Products Rich in Biocompounds
Source: Plant Foods Hum Nutr. 2016 Apr 1;71:165–73. doi: 10.1007/s11130-016-0539-4 (PMC4891392; doi:10.1007/s11130-016-0539-4)
Supplement: Supplementary file 1 — (DOCX 23 kb) [file 11130_2016_539_MOESM1_ESM.docx]

**ELECTRONIC SUPPLEMENTARY MATERIAL OF THE ARTICLE:**

**Physical and bioactive properties of muffins enriched with raspberry and cranberry pomace powder: A promising application of fruit by-products rich in biocompounds**

Sylwia Mildner-Szkudlarz^a^^[[1]](#footnote-1)^, Joanna Bajerska^b^, Paweł Górnaś^c^, Dalija Segliņa^c^, Agnieszka Pilarska^a^, Teofil Jesionowski^d^

^a^[Institute of Food Technology of Plant Origin](http://www.au.poznan.pl/itzpr/), Poznań University of Life Sciences, Poland,

^b^Department of Human Nutrition and Hygiene, Poznań University of Life Sciences, Poland,

^c^Latvia State Institute of Fruit‐Growing, Dobele, Latvia,

^d^Institute of Chemical Technology and Engineering, Poznan University of Technology, Poznań, Poland

| Table A. The chemical characteristics of raspberry and cranberry pomaces | | |
| --- | --- | --- |
|  | **Raspberry pomace** | **Cranberry pomace** |
| moisture | 4.5±0.08 | 7.2±0.10 |
| protein (%) | 7.4±0.12 | 6.8±0.15 |
| fat (%) | 6.6±0.15 | 6.1±0.24 |
| ash (%) | 2.0±0.00 | 0.9±0.01 |
| total carbohydrate (%) | 79.5±0.41 | 79.0±0.55 |
| insoluble DF (%) | 72.2±0.99 | 66.3±0.87 |
| soluble DF (%) | 2.2±0.02 | 5.6±0.05 |
| fructose (%) | 11.6±0.15 | 1.9±0.05 |
| glucose (%) | 9.2±0.12 | 6.7±0.11 |
| WHC (g H2O/g solid) | 7.4±0.06 | 15.7±0.21 |
| OHC (g oil/g solid) | 1.0±0.07 | 1.4±0.08 |

| Table B. Significance of main effects and their interactions on mean percent recovery of main tocopherols (T) and tocotrienols (Tt_3_) of raspberry (RP) and cranberry (CP) pomaces after baking in a model muffin system | | | | | | | |
| --- | --- | --- | --- | --- | --- | --- | --- |
|  | RP-formulated muffins | | | CP-formulated muffins | | | |
|  | α-T | γ-T | δ-T | α-Tt_3_ | γ-Tt_3_ | α-T | γ-T |
| *L, P* value | < 0.001 | < 0.001 | < 0.05 | < 0.001 | < 0.05 | NS | < 0.001 |
| *Bc, P* value | < 0.001 | < 0.001 | < 0.001 | < 0.001 | < 0.001 | NS | < 0.001 |
| *P* interaction (*L×Bc*) | < 0.001 | < 0.05 | NS* | NS | NS | NS | NS |

Values are mean ± SDs of three determinations

*L*: the level of pomaces, *Bc*: baking conditions, *L* × *Bc:* the interaction between these factors

*Not significant, P > 0.05

Table C. Significance of main effects and their interactions on mean percent recovery of polyphenolic compounds of raspberry (RP) and cranberry (CP) pomaces after baking in a model muffin system

|  | RP-formulated muffins | | | | | | |
| --- | --- | --- | --- | --- | --- | --- | --- |
|  | cy-3-soph | cy-3-glc-rut | cy-3-glc | cy-3-rut | qrc-3-ara | qrc-3-glr | ellagic acid |
| *L, P* value | NS* | NS | < 0.05 | NS | NS | < 0.05 | < 0.001 |
| *Bc, P* value | < 0.001 | < 0.001 | < 0.001 | < 0.001 | NS | NS | < 0.001 |
| *P* interaction (*L×Bc*) | < 0.001 | NS | < 0.05 | < 0.05 | NS | NS | NS |

|  | CP-formulated muffins | | | | | | | | | | | |
| --- | --- | --- | --- | --- | --- | --- | --- | --- | --- | --- | --- | --- |
|  | cy-3-gal | cy-3-ara | peo-3-gal | peo-3-ara | myr-3-gal | myr-3-ara | qrc-3-gal | qrc-3-glc | qrc-3-xyl | qrc-3-ara | qrc-3-rham | syr-3-gal |
| *L, P* value | < 0.001 | < 0.001 | < 0.001 | < 0.05 | NS | NS | NS | < 0.05 | NS | NS | NS | NS |
| *Bc, P* value | < 0.001 | < 0.001 | < 0.001 | < 0.001 | NS | NS | NS | < 0.05 | NS | NS | NS | NS |
| *P* interaction (*L×Bc*) | < 0.01 | < 0.01 | < 0.01 | NS | NS | NS | NS | NS | NS | NS | NS | NS |

*L*: the level of pomaces, *Bc*: baking conditions, *L* × *Bc:* the interaction between these factors

Sample codes: M0 – control muffins, RP10 and RP20 – muffins with 10 and 20% raspberry pomace addition, CP10 and CP20 muffins with 10 and 20% cranberry pomace addition. Phenolic compounds: cy-3-soph – cyanidin-3-*O*-sophoroside, cy-3-glc-rut – cyanidin-3-*O*-glucosyl-rutinoside, cy-3-glc – cyanidin-3-*O*-glucoside, cy-3-rut –cyanidin-3-*O*-rutinoside, qrc-3-ara – quercetin-3-*O*-arabinoside, qrc-3-glr –quercetin-3-*O*-glucuronide, cy-3-gal – cyanidin 3-*O*-galactoside, cy-3-ara – cyanidin 3-*O*-arabinoside, peo-3-gal – peonidin 3-*O*-galactoside, peo-3-ara – peonidin 3-*O*-arabinoside, myr-3-gal – myricetin 3-*O*-galactoside, myr-3-ara – myricetin 3-*O*-arabinoside, qrc-3-gal – quercetin 3-*O*-galactoside, qrc-3-glc – quercetin 3-*O*-glucoside, qrc-3-xyl – quercetin 3-*O*-xyloside, qrc-3-rham – quercetin 3-*O*-rhamnoside, syr-3-gal – syringetin 3-*O*-galactoside

*Not significant, P > 0.05

1. Corresponding author: Sylwia Mildner-Szkudlarz, Department of Food Science and Nutrition, [Institute of Food Technology of Plant Origin](http://www.au.poznan.pl/itzpr/), Poznań University of Life Sciences, Wojska Polskiego 28, 60-637 Poznań, Poland; tel.: (4861) 848 72 72; fax: (4861) 848 73 14; e-mail: mildners@up.poznan.pl [↑](#footnote-ref-1)
